# Detection of Composite Communities in Multiplex Biological Networks

**DOI:** 10.1038/srep10345

**Published:** 2015-05-27

**Authors:** Laura Bennett, Aristotelis Kittas, Gareth Muirhead, Lazaros G. Papageorgiou, Sophia Tsoka

**Affiliations:** 1 Centre for Process Systems Engineering, Department of Chemical Engineering,University College London, Torrington Place, London WC1E 7JE, United Kingdom; 2 Department of Informatics, Faculty of Natural and Mathematical Sciences, King's College London, Strand, London WC2R 2LS, UnitedKingdom

## Abstract

The detection of community structure is a widely accepted means of investigating the principles governing biological systems. Recent efforts are exploring ways in which multiple data sources can be integrated to generate a more comprehensive model of cellular interactions, leading to the detection of more biologically relevant communities. In this work, we propose a mathematical programming model to cluster multiplex biological networks, i.e. multiple network slices, each with a different interaction type, to determine a single representative partition of composite communities. Our method, known as SimMod, is evaluated through its application to yeast networks of physical, genetic and co-expression interactions. A comparative analysis involving partitions of the individual networks, partitions of aggregated networks and partitions generated by similar methods from the literature highlights the ability of SimMod to identify functionally enriched modules. It is further shown that SimMod offers enhanced results when compared to existing approaches without the need to train on known cellular interactions.

Cellular organisation is assumed to be modular^1^, with each module driving a distinct biological process. This topology is known as community structure^2^ and its detection is widely accepted as a means of revealing the relationship between topological and functional features of biological systems^3^. Communities, also known as modules, have been shown to comprise groups of biomolecules that physically interact, are functionally cohesive, co-regulated or correspond to biological pathways^4^. Community detection applications have linked molecular compounds with disease^5^, correlated the organisation of cancer signalling networks with patient survival rate^6^ and identified functional modules related to coronary artery disease^7^.

Applications of community structure detection to biological systems often consider networks of a *single* interaction type. However, biological processes are realised via a variety of mechanisms. Biological interactions may be physical or genetic, they may be protein-protein or protein-DNA interactions or describe cellular signalling, regulation of gene expression or the biochemical reactions of metabolic pathways. Each interaction type represents a different aspect of cellular activity and therefore, modules corresponding to cellular functions may be better represented by multiple interaction sources^8^. Consequently, community structure detection has been explored within the context of multiplex networks, i.e. networks with edges that are categorised according to type, sometimes known as multi-dimensional, multi-layer or multi-slice networks^9^, where each edge type is associated to an individual network slice or layer.

Modules comprising more than one interaction type are known as *composite* modules^4^. Algorithms that identify composite modules may help to address various issues associated with analysis of biological data. For example, high-throughput techniques often exhibit biases and datasets corresponding to specific interaction type may have limited coverage. Therefore, it makes sense to combine data from various sources, so as to reinforce true positive interactions and uncover a more representative picture of the underlying biology. Furthermore, there is currently a large number of publicly available resources which archive diverse biological associations^10^. It therefore makes sense to capitalise on such readily available information to build a broader description of cellular interactions.

With regards to existing methods that target composite module detection, two models have been proposed to derive composite modules specifically from physical and genetic interactions. The between-pathway model searches for communities where physical interactions occur inside a module and genetic interactions connect different modules, whereas the within-pathway model searches for modules containing both physical and genetic interactions^11^. It was later proposed that information about within- and between-module interactions can be learned from biological data^12^. Similar methods are also described elsewhere^13^^,^^14^.

More generally, network aggregation methods combine network slices to generate a single network and then standard clustering methods can be used to identify communities^8^^,^^15^^,^^16^. Alternatively, partitions of individual slices can be combined to produce a single partition, i.e. consensus clustering^17^,^18^,^19^. Finally, the modularity metric has been modified to address multiplex networks^20^, where the original definition of modularity for community detection^21^ is altered so that network slices are coupled by linking nodes in one slice to themselves in other slices. A higher degree of coupling forces nodes to belong to the same community across slices, thereby producing a single partition.

Here, we aim to extend the original definition of the modularity metric^21^ to develop an approach to partitioning biological multiplex networks, without restrictions on interaction type, number of network slices or the need to train on known biological data, all features of the methods discussed previously^11^^,^^12^^,^^22^. We report a mixed integer non-linear programming (MINLP) model, SimMod, which takes multiple network slices as input, optimises average modularity across all slices and returns a single partition of composite communities. The procedure is outlined in Fig. 1. SimMod is evaluated through application to yeast networks of physical, genetic and co-expression interactions, as well as through comparisons with other methods that deal with composite module detection and multiplex networks.

## Methods

### A mathematical programming model for clustering multiple network slices

Mathematical programming provides a flexible and intuitive option for the partitioning of biological networks and has been shown to be competitive in numerous community detection algorithms,^23^,^24^,^25^,^26^,^27^,^28^,^29^. Here we extend our previous work^23^,^24^,^25^ and report an MINLP model that, given a multiplex network of two or more slices, optimises average modularity across all slices and returns a single partition. This approach, known as SimMod, is outlined below:
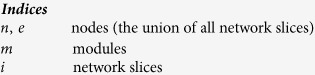

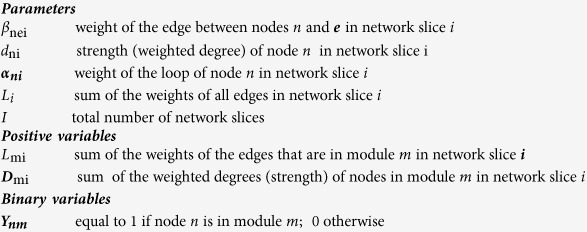


Before defining the objective function employed in SimMod, we first provide the definition of modularity for a single network slice, 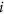:
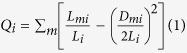
where 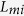 is the sum of the weights of the edges that lie in module 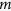, 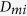 is the sum of the strengths of the nodes that are in module 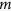 and 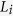 is the sum of the weights of all edges in the network slice 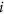. It follows that the average modularity over all network slices can be written as:
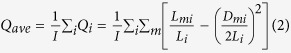

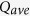
 is maximised subject to the following constraints. First, all modules in the output partition are disjoint, i.e. each node can only be allocated to one module:
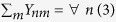


Second, the total degree of module 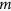 in network slice 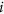, 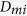, is calculated by:
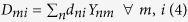
where the strength of a node 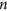 in slice 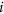 is defined as 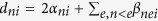. Note that, if 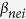 is non-zero, then an edge exists between nodes 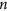 and 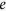 in slice 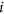 and 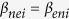.

Finally, an edge is in module 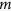 in slice 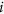 if both of its associated nodes, 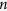 and 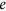, are also in module 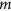. Therefore, the total sum of the weights of all edges in module 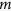 in network slice 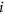, 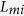, is defined by the following non-linear equality:
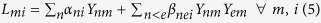


The resulting MINLP model (SimMod) comprises a non-linear objective function with a combination of integer and continuous positive variables. To ensure that we give a reasonable representation of solution space, for each clustering experiment, the MINLP is solved iteratively 100 times, each time with a different random initial. The best partition is taken as the solution with the largest value of 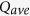. SimMod is implemented in GAMS (General Algebraic Modelling System)^30^ with SBB (standard branch and bound method) mixed integer optimisation solver and CONOPT as the NLP solver with relative and absolute gaps set to zero. Even though, in the case of large networks examples, an upper bound for the number of modules is provided, it is stressed that the actual number of modules in the partition is decided by the model.

### Network datasets

Two yeast interaction datasets were obtained: (i) physical interactions established through two tandem affinity purification followed by mass spectrometry (TAP-MS) datasets and (ii) genetic interactions obtained from an E-MAP screen measuring genetic interactions among genes involved in yeast chromosomal biology^22^. The main connected component of the physical interaction network comprises 784 nodes and 5939 edges, while the genetic interaction network contains 733 nodes and 16864 edges. The union of the two networks gives a set of 1320 nodes, while the intersection comprises 197 nodes. Both networks are weighted, with larger values indicating greater confidence in the interaction. The edge weights of each network were normalised by dividing by the largest edge weight.

A co-expression network was constructed using data representing 44 yeast samples across multiple stages of the cell cycle as described in^31^. Weighted gene co-expression network analysis (WGCNA)^32^ was used to establish the adjacency matrix using soft thresholding, such that the degree distribution satisfied the scale-free topology criterion^33^. A subset of this dataset corresponding to 2728 nodes with the highest variance across samples and 24318 edges was selected. The main component was used in our experiments and consisted of 2578 nodes and 24230 edges. We note that 556 nodes in the co-expression network also appear in the physical and/or genetic networks.

‘Combined’ networks were also constructed, where the individual networks were aggregated into a single network with edge weights equal to the sum of the normalised weights of the respective edges in the individual networks. Aggregating the physical and genetic networks generated a network of 1320 nodes and 22662 edges. Similarly, the weighted union of the physical, genetic and co-expression networks represented 3342 nodes and 46245 edges.

### Comparative analyses

The community structure detected by SimMod across multiple network slices is compared with communities in (i) each network slice individually and (ii) combined networks. Where a clustering method that takes a single network as input is required, we employ Louvain^34^, a well known greedy agglomerative method that optimises modularity (i.e. 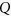 in Equation (1)) with low computational cost and high quality results on large networks. SimMod results are also compared with two methods from the literature: (i) PanGIA^12^, a method specifically designed to partition a two-slice biological network of physical and genetic interactions, and (ii) genLouvain^20^, an extension of modularity optimisation that is applicable to any number of networks slices of any interaction type.

PanGIA carries out logistic regression training on known protein complexes to determine the likelihood of protein pairs belonging to the same module. Unlike SimMod or genLouvain, PanGIA filters nodes so that not all nodes are assigned to a module. A Cytoscape plugin implementation^22^ involves three user-defined parameters: module size, network filter degree and edge reporting. Module size determines if the results will include a larger number of small modules or a smaller number of large modules. For the networks under consideration, the value 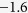 was adopted as in previous studies^22^. The network filter degree parameter determines the extent of node filtering. As SimMod assigns all nodes in all input networks to a module, we leave this parameter blank in order to enforce no filtering, as suggested in the documentation. Edge reporting determines the 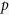-value for which an edge is retained, set to 0.05, as in^22^.

GenLouvain optimises a revised modularity metric^20^ in a two-phase iterative procedure similar to the Louvain method. In genLouvain, a null model is formulated in terms of stability of communities under Laplacian dynamics, incorporating inter-slice connections and a parameter controlling the inter-slice coupling. We note that one does not explicitly define inter-slice connections in the method input file but only a set of interactions categorised by type or time point, if the dataset reflects temporal interactions. In addition, genLouvain involves two user-defined parameters. First, we select the default resolution level, 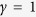. Second, the degree of coupling between network slices, 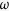, must be defined. The coupling edges have a value of either 0 or 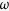, i.e. the corresponding coupling edge either exists, or not. If 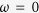, modularity for each network slice is optimised independently generating a partition for each network slice. By assigning a higher degree of coupling, nodes are forced to belong to the same community across slices, producing a single partition and rendering the method comparable to SimMod. In our experiments, 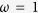 and 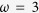 were chosen as they generate a single partition for the two and three-network cases, respectively. The genLouvain method is implemented in Matlab^35^.

### Mutual information

Normalised mutual information (NMI)^36^ is a measure of similarity between two partitions, which ranges from 0 for dissimilar to 1 for identical community structures. This measure is taken from information theory and intuitively shows how much information is shared between two partitions. In cases where partitions do not comprise the same set of nodes, the nodes common to both partitions are included in the mutual information calculation.

### Functional enrichment analysis

Gene Ontology (GO) under the ‘Biological Process’ category has been employed to express the functional content of a node^37^. In order to determine the annotation enrichment of a particular GO term 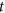 in module 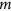 containing 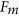 nodes, the probability of the same or higher number of nodes being annotated with this term if 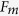 nodes are randomly selected from the network, is calculated^38^. This is a statistical test involving the upper tail of a hyper-geometric distribution, also known as the one-tailed Fisher’s exact test. A disadvantage of this method is the inheritance problem^38^, i.e. a gene which is annotated to 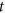 is also annotated to all parent (less specific) terms of 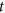. To address this, the *parent-child* method for detecting GO term enrichment is employed^39^. Since the statistical test is performed for multiple GO terms, the 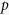-values are adjusted using the Bonferroni-Holm multiple test correction method^40^. In our analysis, a GO term 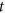 is characterised as *enriched* in module 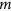 if it has an adjusted 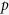-value 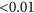, with its enrichment score 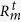 given by:
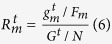
where 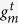 and 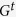 are the numbers of genes annotated with GO term 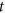 in module 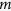 and the whole network, respectively, and 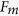 and 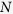 are the number of nodes in module 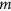 and the entire network, respectively. The enrichment analysis was performed through *Ontologizer*^38^^,^^41^, using yeast GO slim term annotations.

## Results and discussion

The physical and genetic interactions of the two yeast networks can be regarded as complementary, i.e. physical interactions represent direct spatial associations whereas genetic interactions describe similar functional role^42^. Genetic interactions have been shown to correlate with physical networks; in yeast, two proteins found in the same area of a genetic network are likely to physically interact^43^^,^^44^, genes exhibiting similar genetic interaction patterns tend to belong to the same protein complex^44^ and highly connected proteins in the physical network are generally highly connected in the genetic network^45^. We therefore hypothesise that by considering these two complementary interaction types simultaneously, more biologically meaningful communities can be detected than if either network was analysed individually. Furthermore, we investigate whether adding a third network based on correlations between gene expression profiles can improve the functional cohesion of the communities derived.

SimMod is evaluated against (i) PanGIA^12^ and genLouvain^20^, (ii) clustering the ‘combined’ networks and (iii) clustering individual network slices. The results obtained for these comparisons are discussed below with regards to the community structure obtained and the functional enrichment of the various partitions.

### Evaluation of modular structure

#### Composite modules of two interaction types

**Figure 2:**
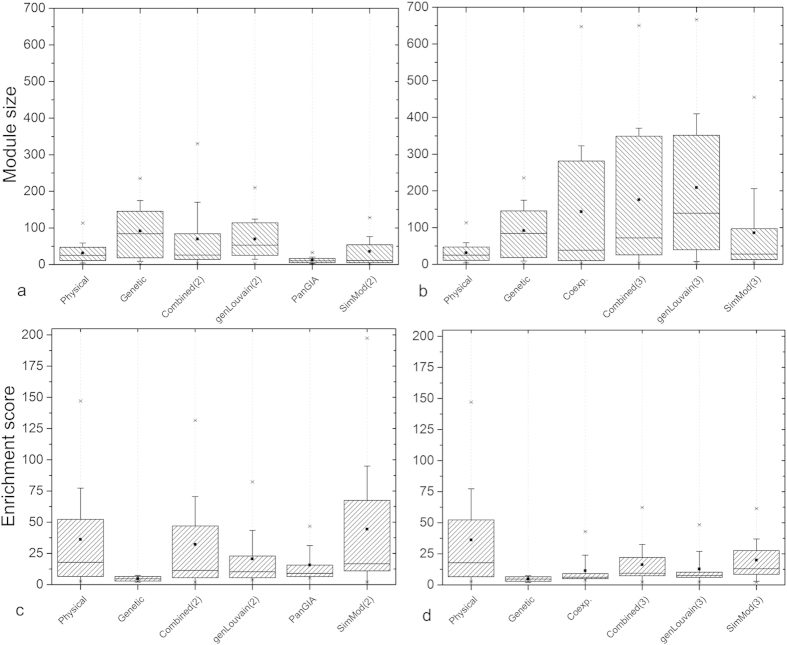
Box plots for (i) module size (**a** and **b**) and (ii) enrichment score (**c** and **d**). Box represents the interquartile range with solid line being the median, small square the mean, and stars minimum/maximum values. Whiskers represent one standard deviation.

SimMod is applied to the yeast physical and genetic interaction networks, denoted SimMod(2). SimMod finds a partition of 37 modules (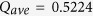); 26 are composite, 10 contain nodes that appear only in the physical network and 1 module comprises nodes that only appear in the genetic network. Module size ranges from 2 to 128 nodes (Fig. 2a). This partition is discussed in the context of various alternative partitions below.

**Figure 3:**
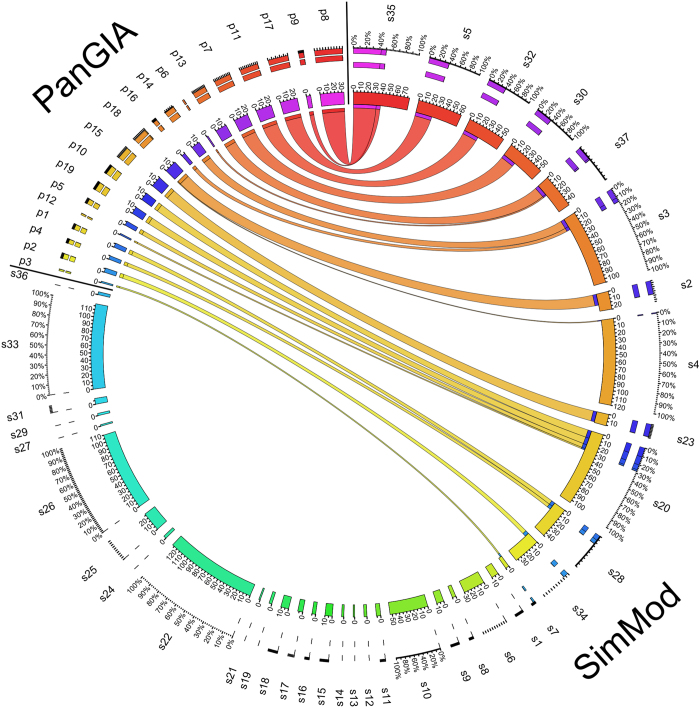
Visual comparison of the modules found by SimMod and PanGIA. Ribbon thickness represents the number of nodes that are common between the corresponding modules. Numbers on coloured segments correspond to the number of nodes that lie within each module. Coloured stripes above the segments show the percentage of coverage between the two methods.

PanGIA clusters the physical and genetic networks and finds a partition of 33 composite modules. Despite leaving the filter degree parameter blank to enforce no filtering, much of the network is left unprocessed with only 234 out of 1320 proteins appearing in the output partition. As mentioned above, PanGIA relies on known molecular interactions of protein complexes to determine composite modules, thereby possibly resulting in high accuracy of the detected composite modules at the cost of lower coverage. PanGIA therefore identifies what can be thought of as ‘benchmark’ composite modules, which SimMod achieves to match without the need of a training set (Fig. 3).

Of the 33 modules found by PanGIA, 14 are singletons and are not considered further in our discussion. The remaining 19 modules contain between 2 and 33 nodes (Fig. 2a). GenLouvain(2) finds a partition of 19 communities, ranging from 3 nodes to 210 nodes (Fig. 2a); 14 communities are composite and 5 contain nodes that are in the physical network only. This is denoted as genLouvain(2).

Despite methodological differences, there is a high level of agreement between with SimMod(2) and PanGIA (NMI equal to 0.585). Specifically, all PanGIA modules match a complete or partial module in SimMod(2), i.e. proteins that belong to the same module according to PanGIA are also found to co-cluster by SimMod, as illustrated in Fig. 3. Six genLouvain modules match a complete or partial module in SimMod(2) and 11 modules have 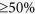 of their nodes in a single module in SimMod(2) (NMI equal to 0.436). GenLouvain also exhibits a fair level of agreement with PanGIA; 15 out of 19 PanGIA modules match a complete or partial module in genLouvain(2) (NMI equal to 0.465).

A limitation of PanGIA is that it specifically accepts a physical interaction network and a genetic interaction network. SimMod does not carry this restriction; the method can be generalised to any interaction type and any number of networks, within computational power allowance. Thus SimMod is more adaptable to different user requirements. In addition PanGIA is sensitive to three user-defined parameters, whereas SimMod does not carry this restriction.

While genLouvain clusters all nodes and carries less restrictions than PanGIA in terms of number of network slices and interaction types, it does require the user to select a value for the coupling parameter, 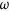. In our experiments, we select the value of 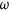 that generates a single output partition, i.e. the same partition for each network slice, thus rendering genLouvain comparable with SimMod. This parameter may have different interpretations for different applications and therefore it may not always be clear which value of 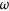 is appropriate. In particular, for biological network applications, this parameter may not be either meaningful or available. On the other hand, such ambiguous parameters do not exist in the SimMod implementation.

Finally we note that both genLouvain and SimMod employ a modified version of the modularity metric. These methods are therefore more similar to each other in computational terms than they are to PanGIA, as they both aim to detect communities by optimising an objective function based on interaction density of the network structure alone. However, while both SimMod(2) and genLouvain(2) correspond well with the ‘benchmark’ modules of PanGIA, mutual information calculations show that SimMod(2) performs more closely to PanGIA. We will investigate whether these results also reflect functional content or if training on biological complexes is indeed required in order to find biologically meaningful modules, as discussed below and shown in Fig. 2c,d.

#### Composite modules of three interaction types

The physical, genetic and co-expression yeast networks are now considered as a multi-slice network where a single partition of composite communities is sought. SimMod (denoted as SimMod(3)) finds a partition of 39 modules, ranging from 3 to 445 nodes (Fig. 2b) with 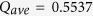. GenLouvain (genLouvain(3)) finds a partition of 16 modules, ranging from 4 to 666 nodes (Fig. 2b). The main difference between SimMod(3) and genLouvain(3) is the number of communities in each partition, 39 and 16, respectively. Furthermore, mutual information shows that SimMod(3) and genLouvain(3) are more dissimilar than SimMod(2) and genLouvain(2) (NMI equal to 0.183 and 0.436, respectively). From topology alone we cannot determine whether the addition of the third network offers improved results over composite modules of two interaction types, but investigate the functional implications below.

#### Clustering aggregated networks

Networks where nodes and interactions are first aggregated into a single network and then clustered, are now discussed. ‘Combined’ networks of two (Combined(2)) and three (Combined(3)) interaction types are partitioned using Louvain^34^. Combined(2) comprises 19 modules (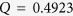), ranging from between 3 and 330 nodes (Fig. 2a) and Combined(3) comprises 19 modules (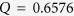), ranging from between 3 and 650 nodes (Fig. 2b). Combined(2) and Combined(3) contain fewer communities than SimMod(2) and SimMod(3), respectively. This suggests that communities are more difficult to identify when networks are aggregated, rather than when optimising modularity simultaneously for all network using SimMod, which preserves the topology of the input networks. Similarly, genLouvain(2) and PanGIA comprise fewer modules than SimMod(2) and genLouvain(3) comprises fewer modules than SimMod(3). Despite not knowing the ‘true’ community structure, from these results one can hypothesise that SimMod may be able to uncover community structure more readily than in methods which tend to aggregate smaller communities. We validate these partitions through functional enrichment analysis as described below.

#### Clustering single interaction type networks

When each interaction type is clustered individually, Louvain detects partitions of 25 modules (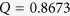), 8 modules (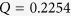) and 18 modules (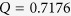) for the physical, genetic and co-expression networks, respectively. The physical network partition contains modules ranging from 3 to 113 nodes, the genetic network comprises modules ranging from 2 to 235 nodes (Fig. 2a) and modules of the co-expression network range from 2 to 647 nodes (Fig. 2b). Using mutual information, we identify the individual networks with the largest ‘influence’ on the various partitions of composite modules.

**Figure 4:**
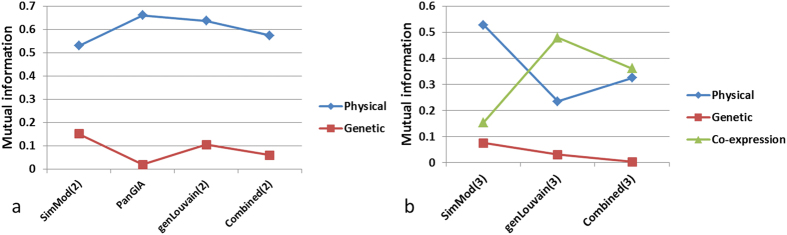
The mutual information between the individual network partitions and the partitions that combine the corresponding networks to produce a partition of composite modules.

Figure 4a shows the mutual information comparisons for any of the partitions combining the yeast physical and genetic networks. In all cases, the partitions of composite modules are markedly more similar to the physical network partition than the genetic network partition. This reflects the difference in strength of community structure exhibited by the individual networks, i.e. all methods appear to be more influenced by the network topology of the physical than the genetic network. When the co-expression network is added (Fig. 4b), the physical network still dominates the SimMod(3) partition, however, less so for genLouvain(3) and combined(3), where the co-expression network appears to have more influence. We investigate this further using GO enrichment analysis, as follows.

### Enrichment analysis of GO terms

GO enrichment analysis, described in the Methods section, is used to evaluate the biological significance of the above results. Fig. 2c–d show box-plots of the enrichment score, 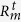, of the term with the highest enrichment in each of the enriched modules in the respective partitions.

When considering only the individual networks, the partition with the highest average enrichment score and the largest percentage of enriched modules arises from the physical network (Fig. 2c), while the less modular topology of the genetic network is reflected in its low enrichment values. Despite the relatively high modular structure of the co-expression network, its partition is less functionally informative than the physical network (Fig. 2d). This is in line with the NMI calculations of the two-network clustering methods (Fig. 4a), i.e. most topological information is captured from the physical network, which is in agreement with its higher functionality. However, each of the three-network approaches react differently to the inclusion of the co-expression network (Fig. 4b). This gives an indication of how each method deals with the inclusion of additional nodes and interactions deriving from the co-expression network, which were not previously included in the physical or genetic networks.

The physical network partition has an average enrichment that is greater than Combined(2), but less than SimMod(2) (Fig. 2c). It appears that simply clustering the combined network does not improve the functional content offered by the single network partition. This may suggest that the aggregated network exhibits a topology that is drastically different from the individual networks and in turn functional properties are lost. On the other hand, SimMod appears to combine the physical and genetic networks in a way that offers a positive effect on the functional content of the composite modules as indicated by enrichment analyses (Fig. 2c). Similarly, SimMod(3) is more functionally informative than Combined(3) (Fig. 2d).

**Figure 5:**
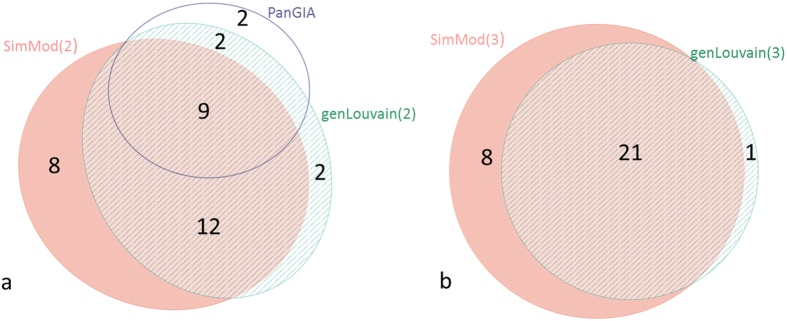
Venn diagram (area proportional) showing common GO terms between (**a**) SimMod(2), PanGIA and genLouvain(2), and (**b**) SimMod(3) and genLouvain(3).

SimMod(2) finds more strongly enriched composite modules as well as an overall greater average enrichment than all other two-network approaches, including genLouvain(2) and PanGIA. Furthermore, SimMod(2) finds a better coverage of the Gene Ontology than PanGIA or genLouvain(2) (Fig. 5a). In the case of PanGIA, this may be partially attributed to the fact that a large portion of the nodes are disregarded, potentially losing functionally important nodes. Both SimMod(2) and genLouvain(2) comprise modules that correspond relatively well with those found by PanGIA (NMI equal to 0.585 and 0.465, respectively), while also including additional nodes that cover the union of both networks. The inclusion of these extra nodes is beneficial as both SimMod(2) and genLouvain(2) exhibit better functional enrichment than PanGIA (Fig. 2c). Therefore, despite PanGIA training on known biological complexes, SimMod and genLouvain highlight the efficacy of searching for composite modules based on interaction density alone.

The addition of the co-expression network affects the performance of both genLouvain and SimMod, reducing the average enrichment and the fraction of enriched modules (Fig. 2d). In particular, the physical network alone offers better functional enrichment than all three-network approaches. This is possibly due to noise added by the co-expression network, which ‘dilutes’ the enrichment information of the derived communities. In contrast, while the genetic network offers low enrichment, SimMod still manages to produce a partition with higher average enrichment due to the dominance of the physical network in the resulting partition (Fig. 2c).

However, we note that SimMod(3) yields a partition with better average enrichment than Combined(3) or genLouvain(3) (Fig. 2d). SimMod(3) also offers a larger coverage of the Gene Ontology than genLouvain(3) (Fig. 5b). NMI calculations (Fig. 4b) show that SimMod finds a partition more similar to the physical network partition than the co-expression partition, while the opposite is true for genLouvain(3) and Combined(3). Thus, it appears that SimMod is less sensitive to the noise of the co-expression network and is able to recover the more functionally informative partition of the physical network. Overall, these results highlight that while combining different interaction types can lead to more biologically relevant results, one must combine data types with an appropriate rationale.

**Figure 6:**
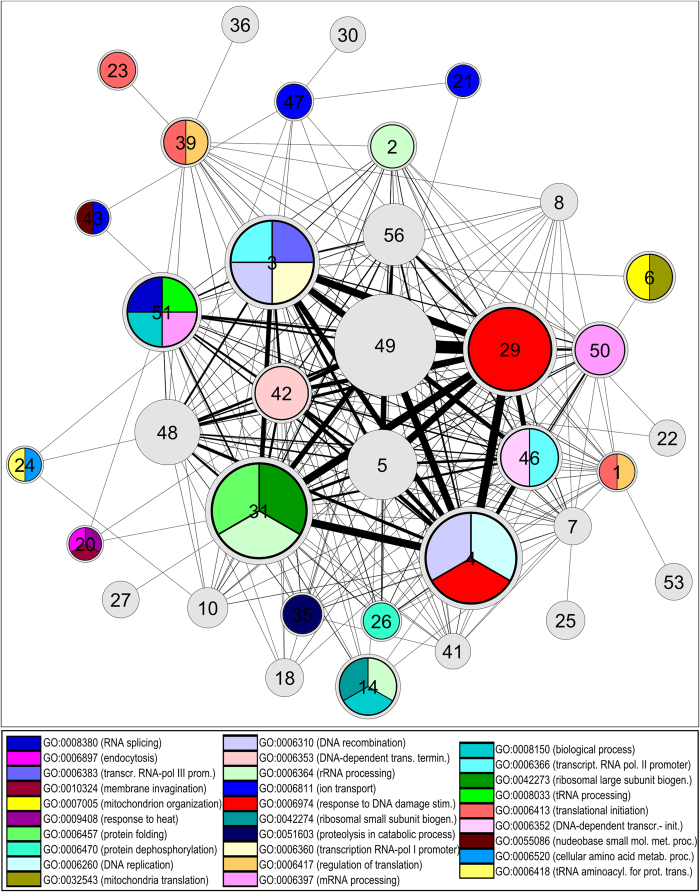
Module network for the SimMod partition of the physical and genetic network. Each node represents a module and its size is proportional to the number of nodes it contains. Edge thickness is proportional to the number of links between different modules. GO terms that are enriched within each module are shown with their corresponding colours. Grey modules are the ones that do not contain any enriched GO terms.

In Fig. 6 we show a representation of the functional repertoire of the modules discovered with SimMod, as a network where each node represents as a community and edges show the interactions that exist between communities, weighted according to the number of interactions. The diameter of nodes is proportional to the size of the corresponding module and the thickness of edges is proportional to the weight of that edge. Each node is coloured according to the enriched GO terms for each module.

Large, as well as small, modules are discovered with specific functionality, e.g. module 29 and module 23, responsible for response to DNA damage stimulus and translational initiation respectively. Other modules are enriched with GO terms of similar functionality, e.g. module 31 comprising ribosome-related functionality, module 20 including membrane related processes, such as invagination and endocytosis, and module 1 and 39 for translation initiation. Module 4 is responsible for response to DNA damage, DNA replication and DNA recombination and strongly linked with module 31 (ribosome related) hinting at the well-known strong connection of biological processes relating to DNA replication and translation. Module 3 is responsible for transcription from RNA Polymerase I-III promoters and DNA recombination and module 6 for the organization of the mitochondrion, as well as mitochondrial translation. Overall, it is argued that SimMod discovers a wide repertoire of functionality organised into modules of specific and inter-related biological processes.

## Conclusions

This work reports a mathematical programming method, SimMod, which clusters multiplex networks and identifies a single partition of composite modules. It is found that clustering network slices using SimMod, rather than simply clustering their aggregation, is a more effective approach towards detecting composite modules. Thus, highlighting the need for more sophisticated means of integrating multiple interaction types into community structure detection algorithms.

SimMod finds modules with a higher average functional enrichment than the other two-network approaches presented. While PanGIA may find high confidence modules due to learning from known protein complexes, both SimMod and genLouvain find more functionally cohesive modules when considering network structure and interaction density only.

As mentioned previously, SimMod and genLouvain are more similar in terms of their modelling approach, as they both optimise variations of the modularity metric. While SimMod achieves this goal by averaging standard modularity across network slices, genLouvain employs a version of modularity where the null model incorporates inter-slice connections. Although the latter may be a more explicit procedure, the inclusion of inter-slice connections may pose several disadvantages. First, within our modelling framework, this objective function would result in a more computationally expensive problem, resulting in scalability restrictions and limited applicability. Second, as mentioned above, the strength of coupling between slices may not be meaningful for all applications, more so in biological networks where such information is not available. We also add that genLouvain tackles a different problem statement by allowing different output partitions for each slice according to the strength of coupling. This is not the aim of SimMod, although this is a direction we can explore in future work. Finally, we note that our less computationally restrictive approach does indeed produce meaningful results across various applications.

It is also demonstrated that while in some cases combining interaction types can improve functional content of the community structure, in other cases the inclusion of noise will dilute functional information. Thus, an appropriate rationale needs to be expended so as to integrate original datasets meaningfully, pertinent to the problem at hand. However, when experiment-specific data is integrated with the appropriate rationale, SimMod has the potential to discover a wide variety of functionally enriched composite modules which can lead to the generation of biological hypotheses relating to particular clustering experiments

Overall, this work offers advances against previous methods that cluster multiplex biological networks, as well as novel application of modularity maximisation principles. Future work on other systems and data sources is intended to illustrate the use of mathematical programming principles for data integration applications.

## Additional Information

**How to cite this article**: Bennett, L. *et al.* Detection of Composite Communities in Multiplex Biological Networks. *Sci. Rep.* **5**, 10345; doi: 10.1038/srep10345 (2015).

## Figures and Tables

**Figure 1:**
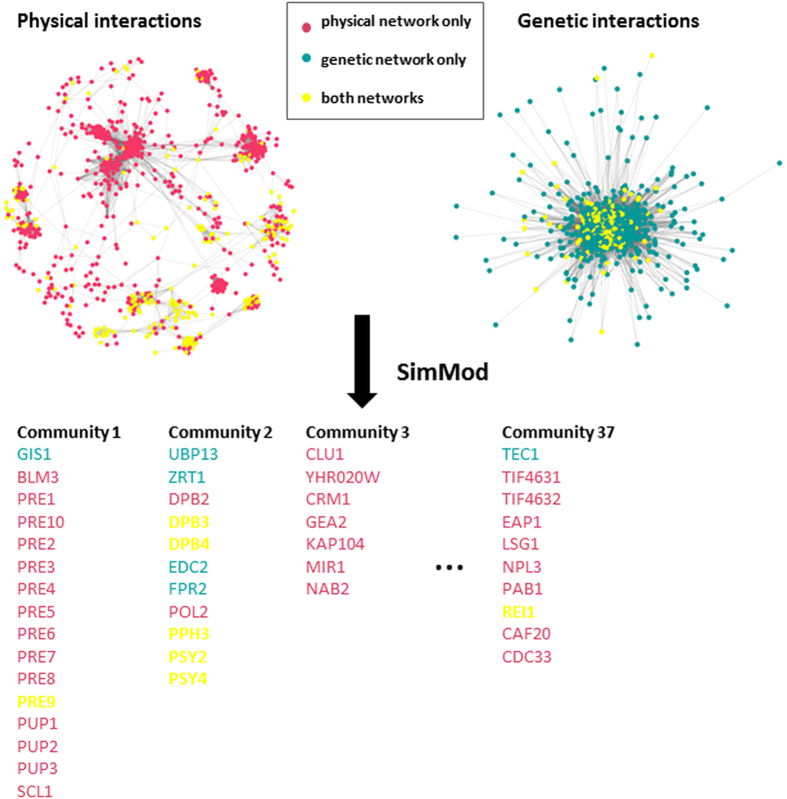
Procedure outline: community structure detection in multiple networks, each with a different interaction type. Two yeast network slices, one with physical and the other and genetic interactions, are visualised and the nodes common to both networks are highlighted in yellow. SimMod clusters these networks and a partition of composite communities is returned.
